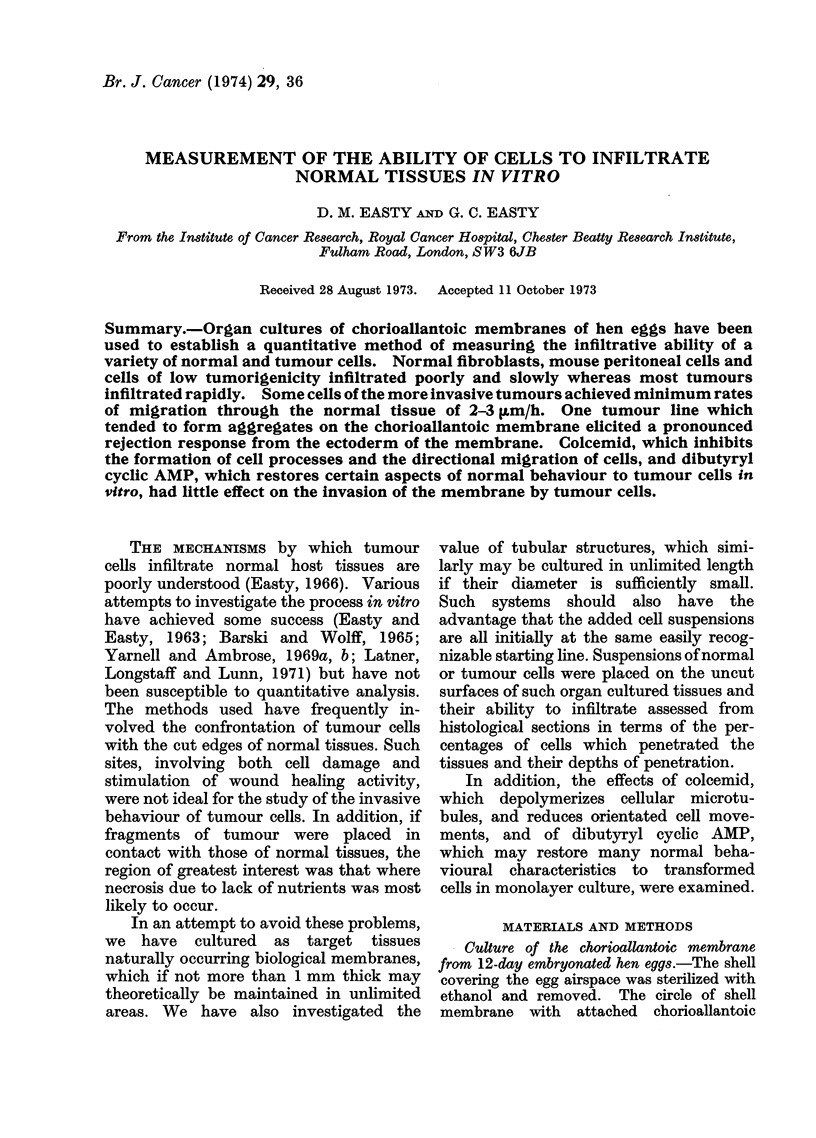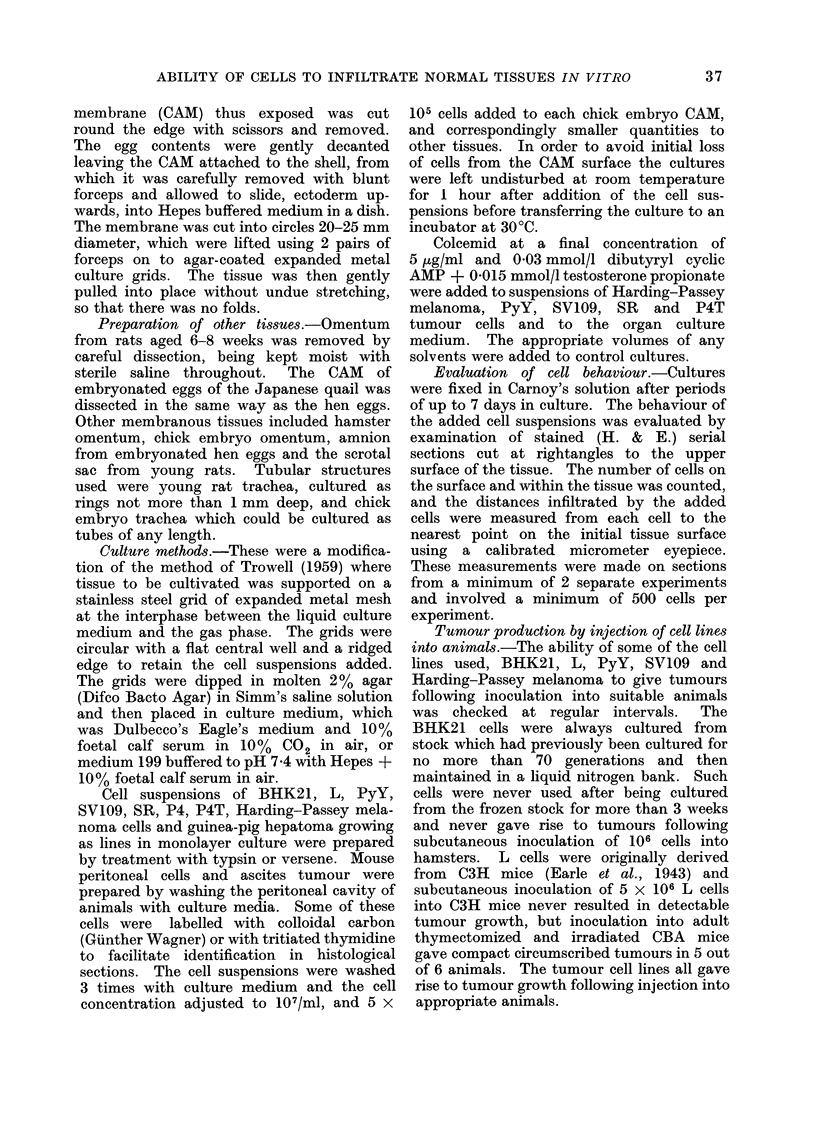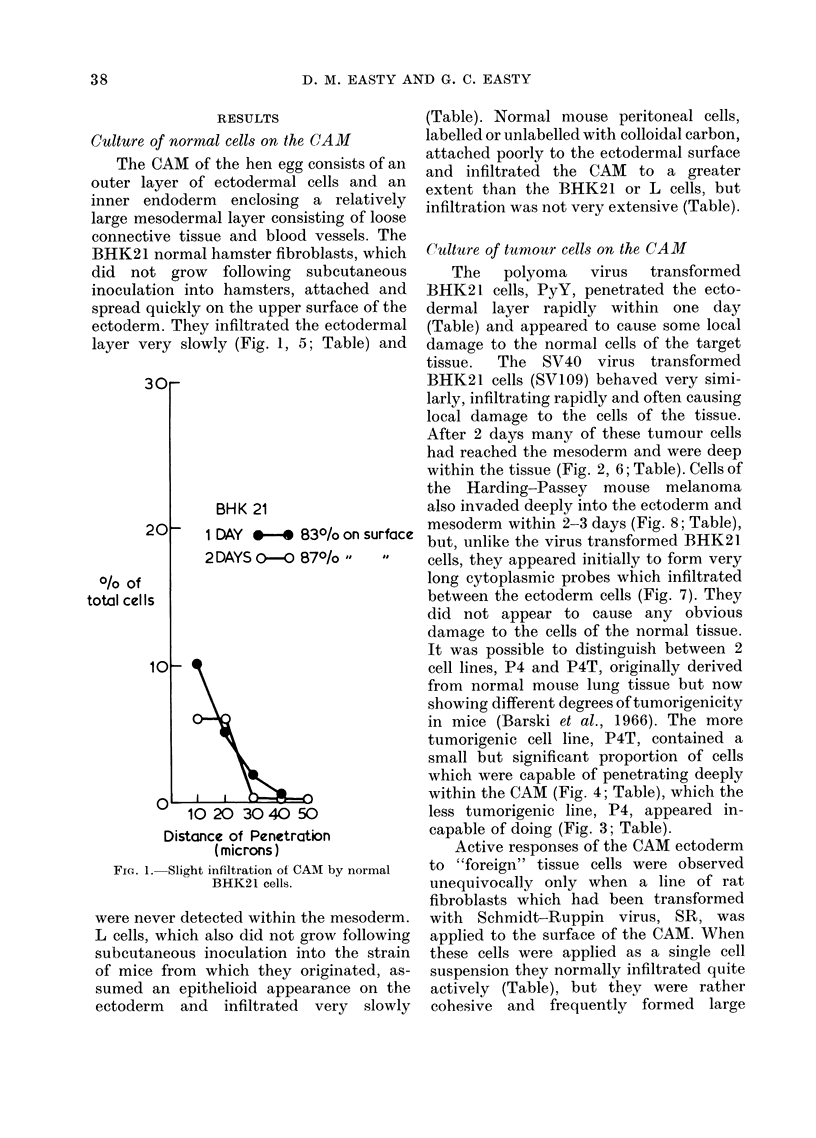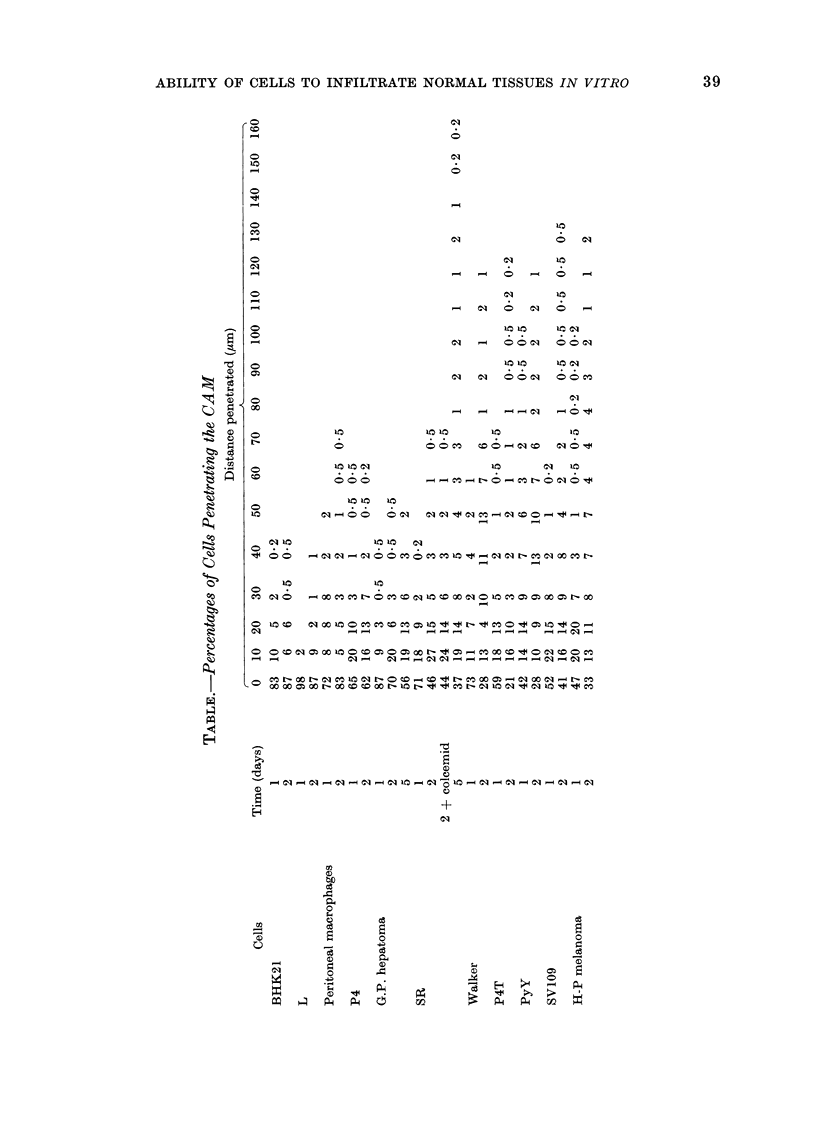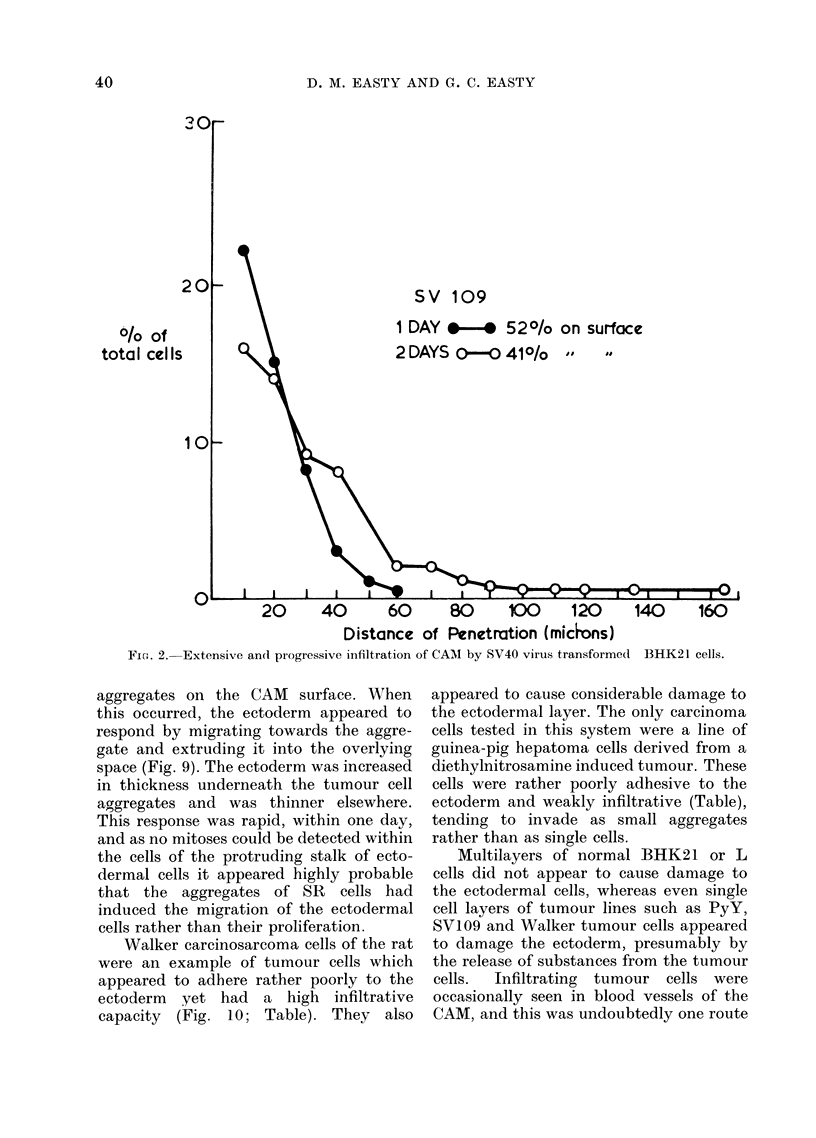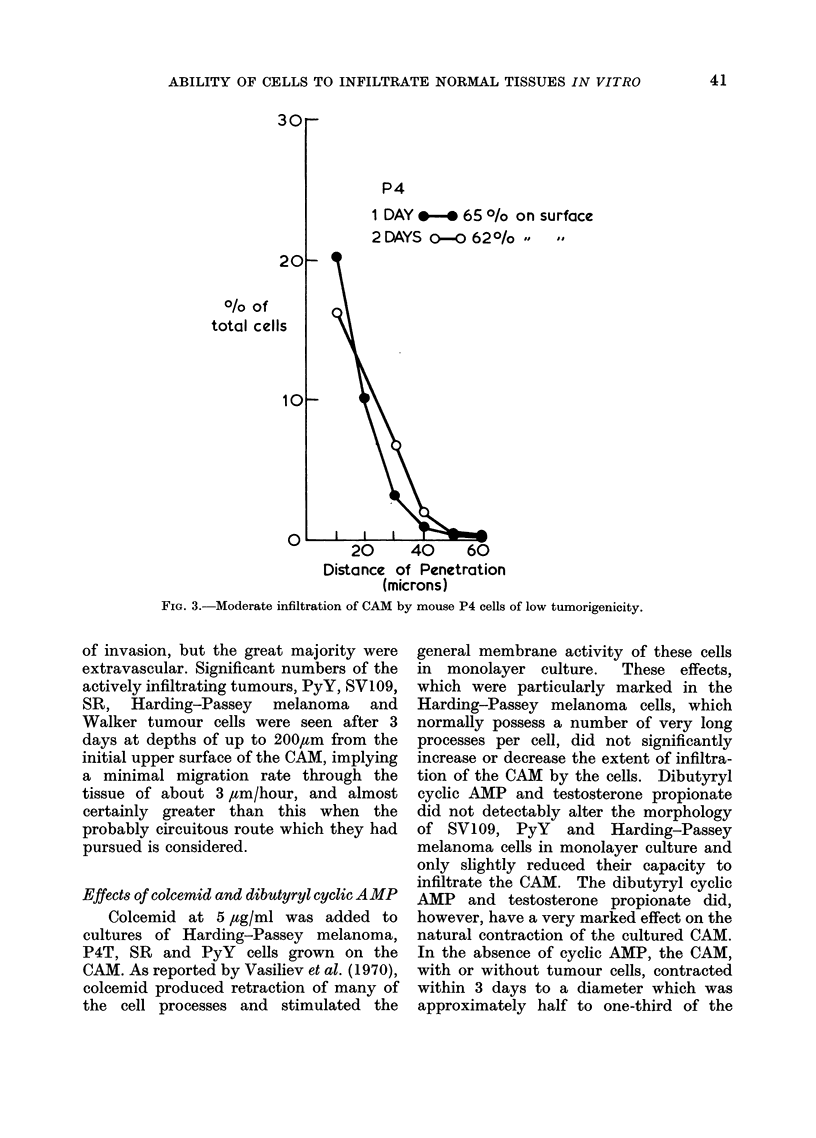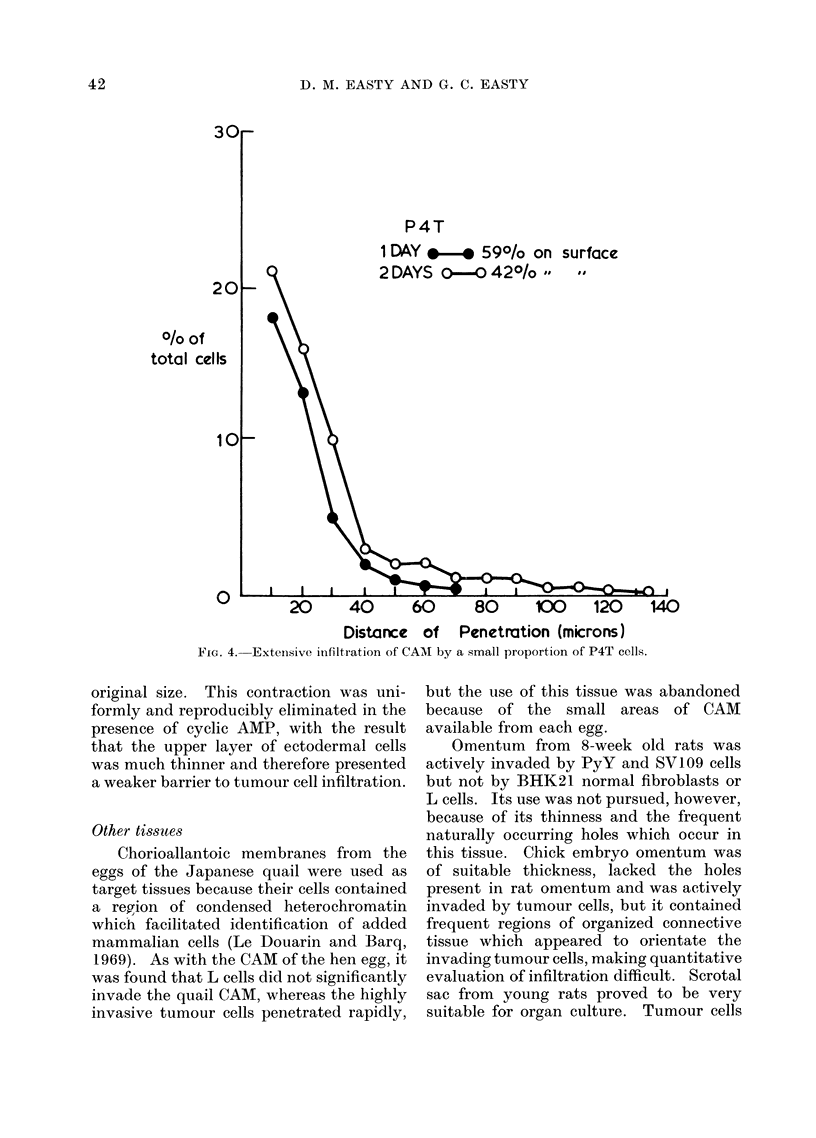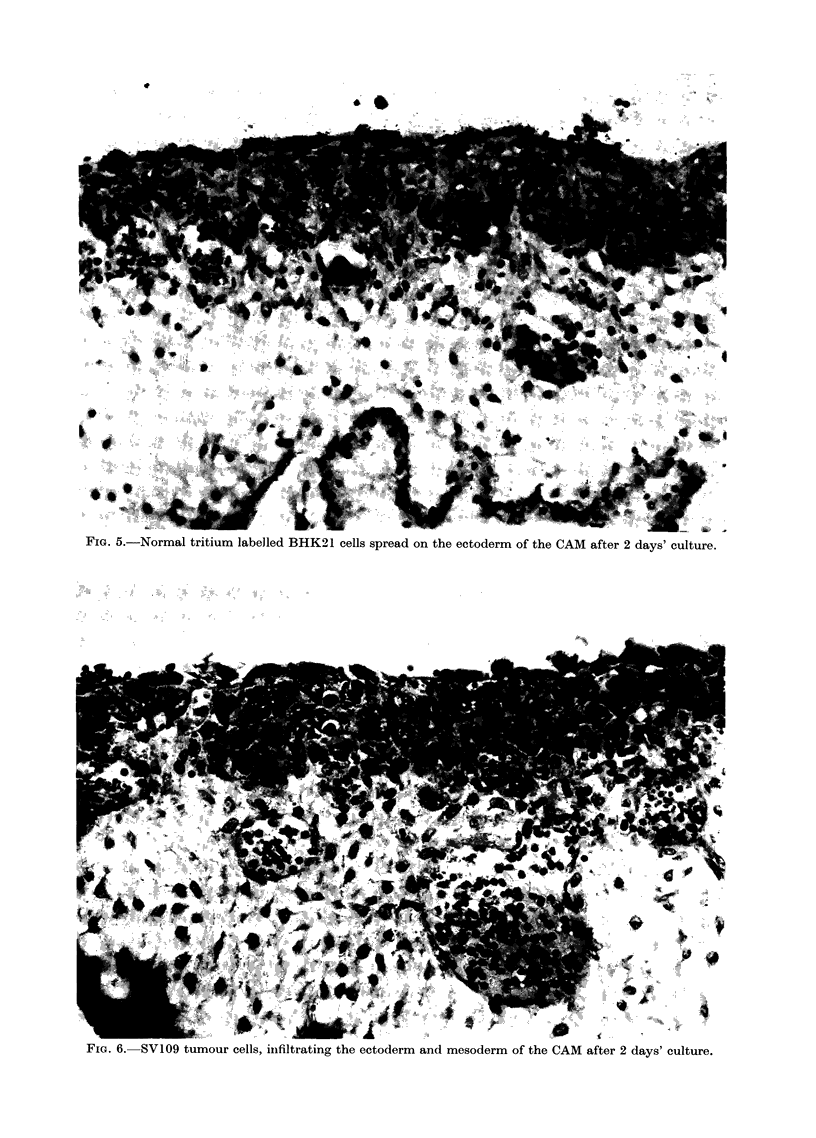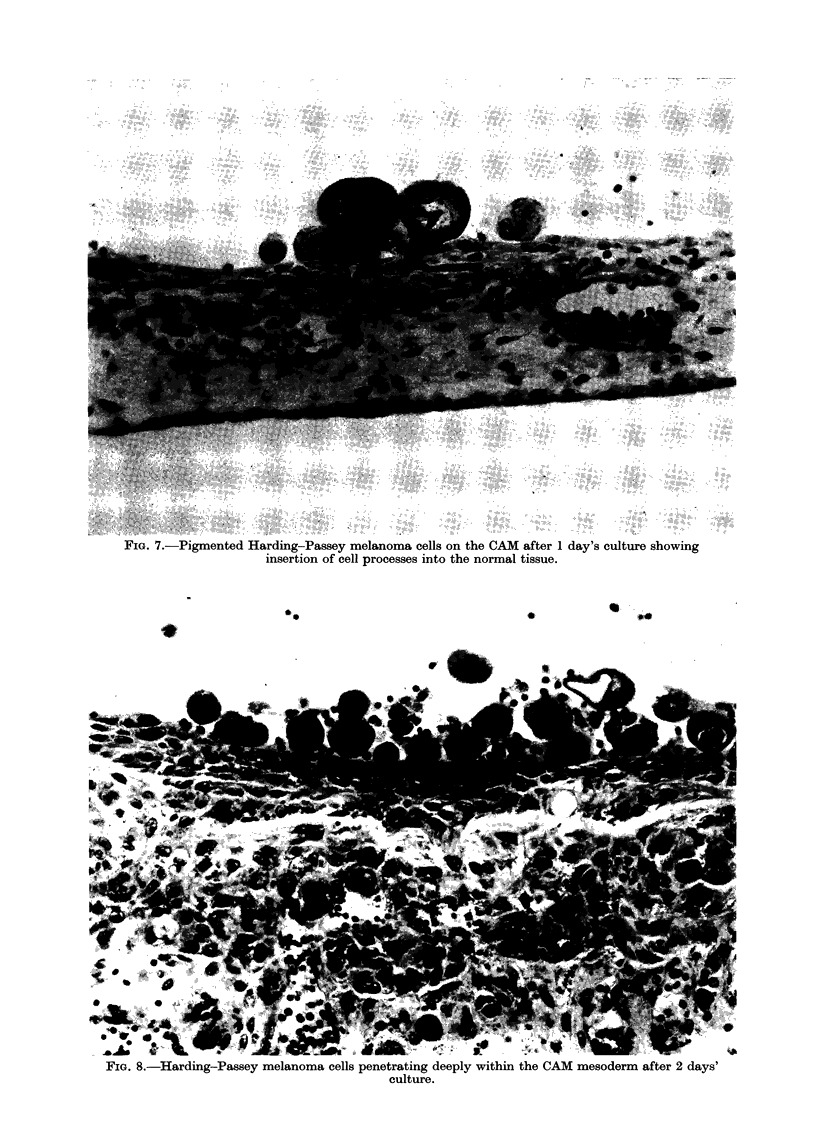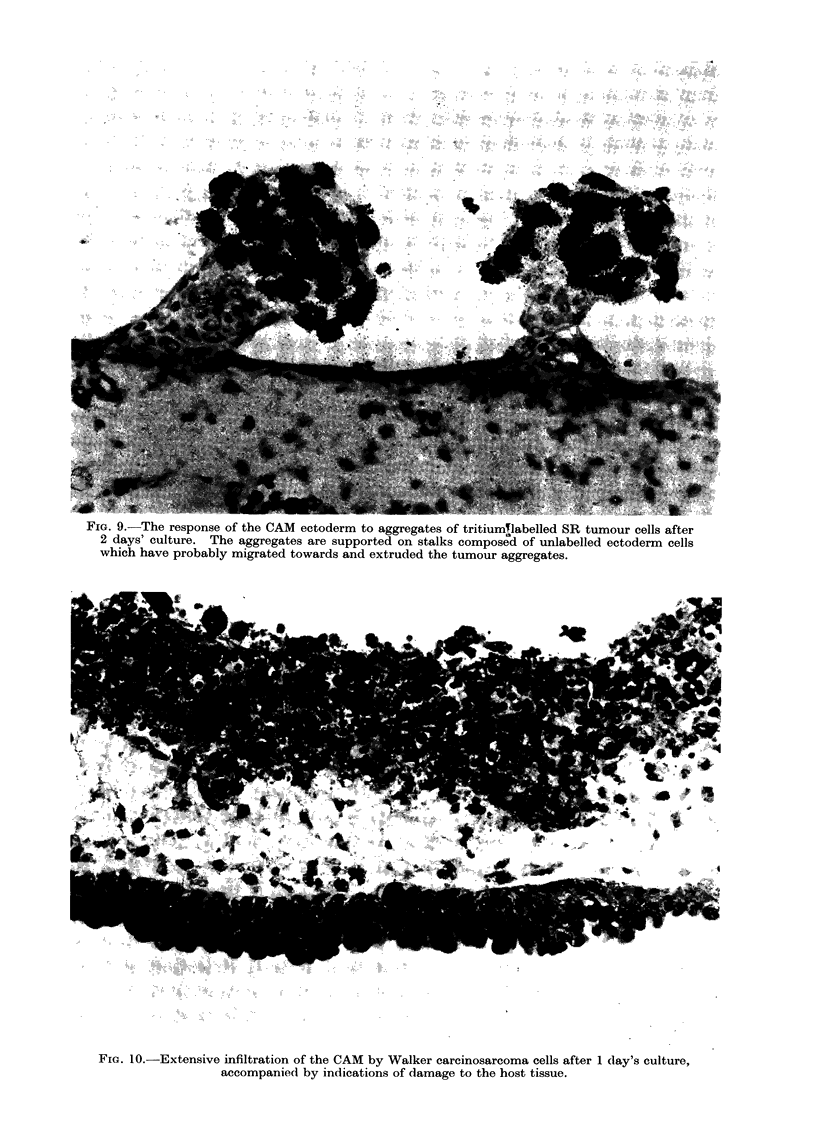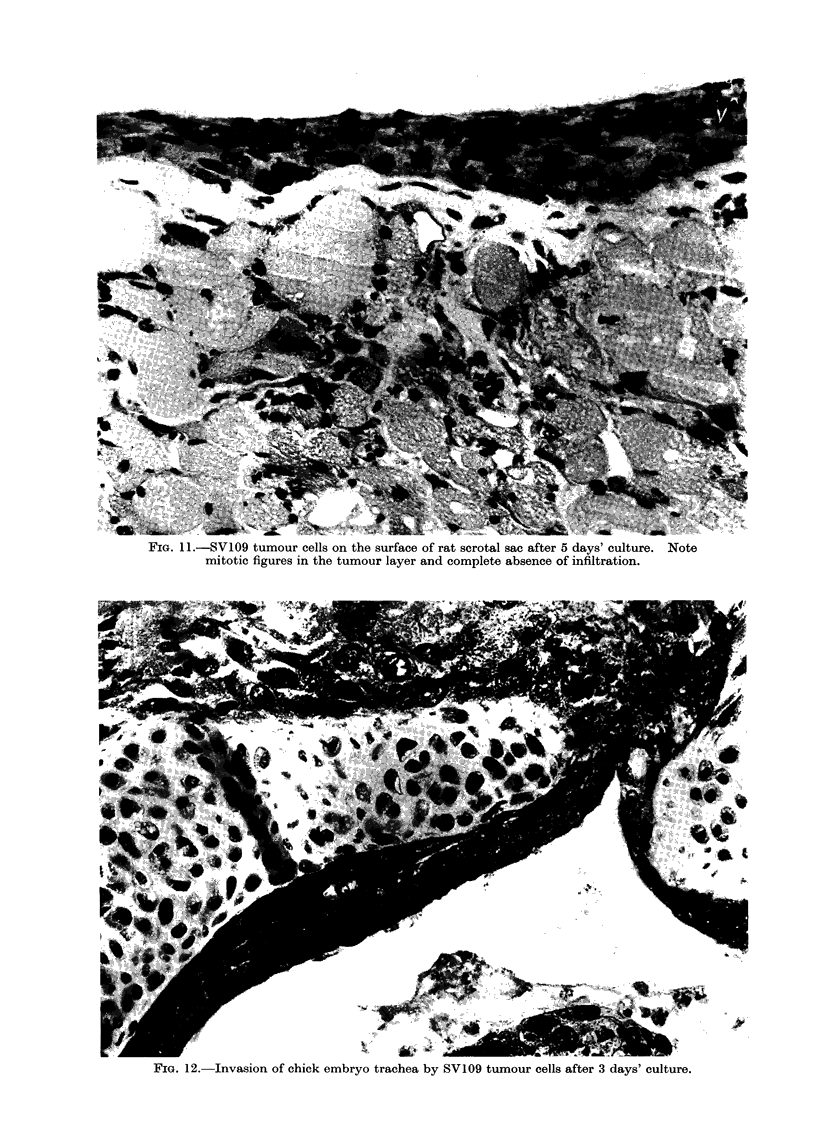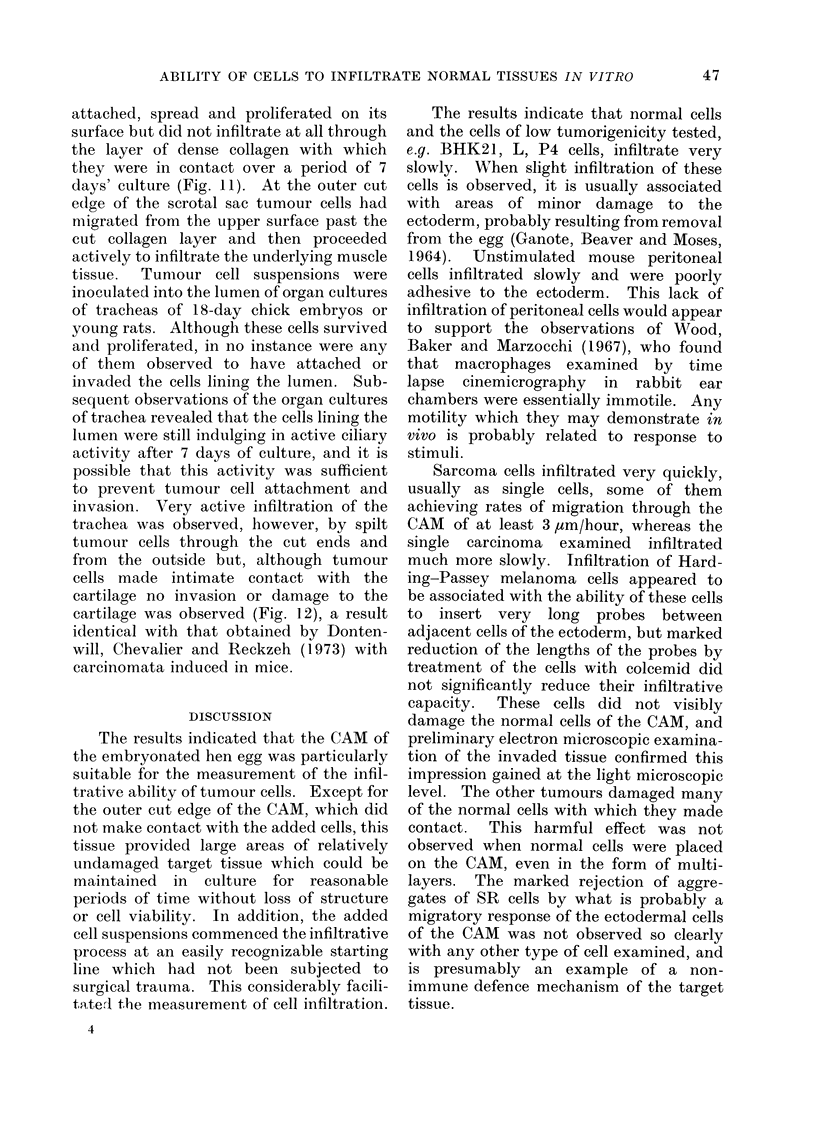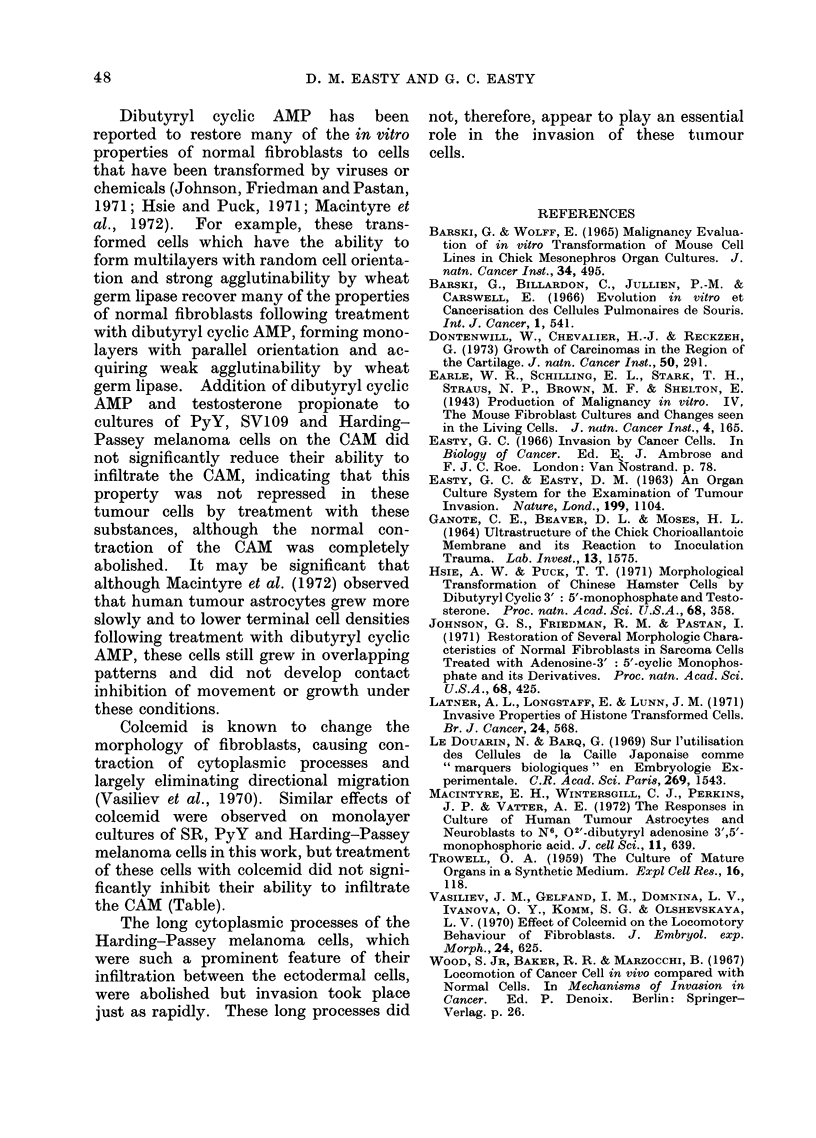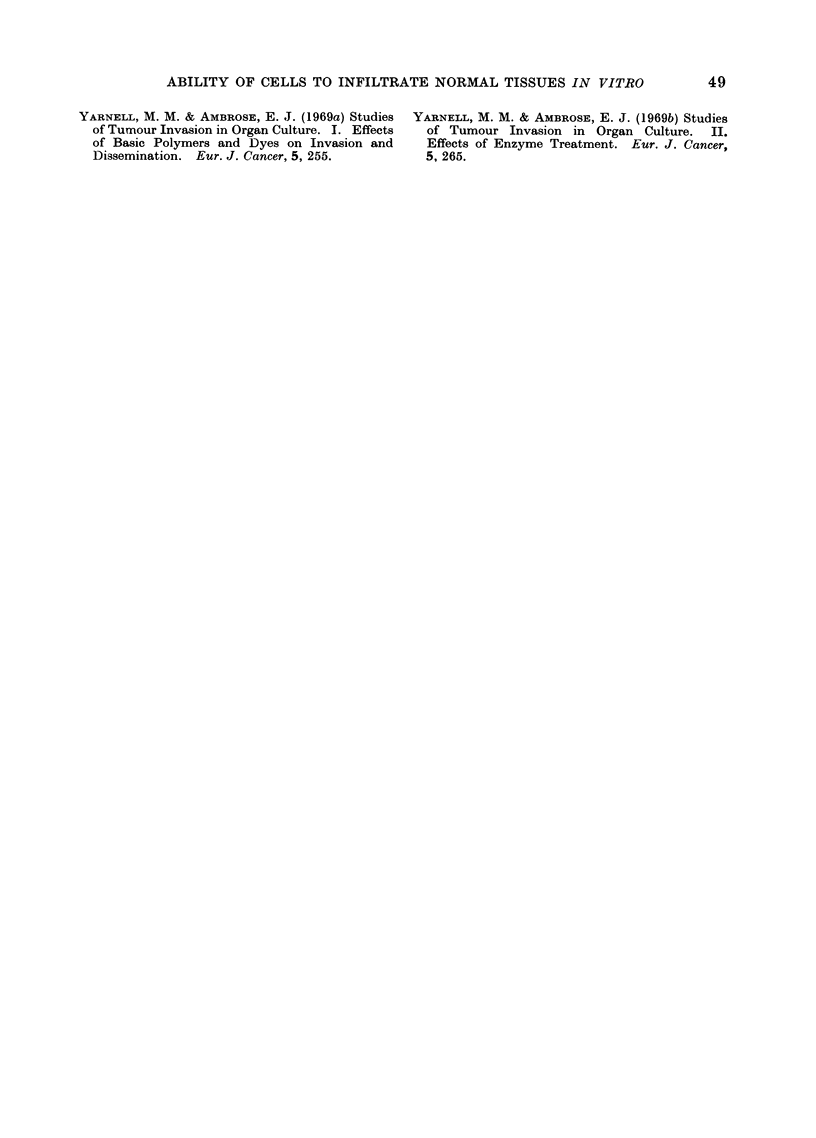# Measurement of the Ability of Cells to Infiltrate Normal Tissues In vitro

**DOI:** 10.1038/bjc.1974.5

**Published:** 1974-01

**Authors:** D. M. Easty, G. C. Easty

## Abstract

**Images:**


*Br. J. Cancer* (1974) **29**, 36

### MEASUREMENT OF THE ABILITY OF CELLS TO INFILTRATE NORMAL TISSUES *IN VITRO*

D. M. EASTY AND G. C. EASTY

*From the Institute of Cancer Research, Royal Cancer Hospital, Chester Beatty Research Institute, Fulham Road, London, SW3 6JB*

Received 28 August 1973. Accepted 11 October 1973

**Summary.—Organ cultures of chorioallantoic membranes of hen eggs have been used to establish a quantitative method of measuring the infiltrative ability of a variety of normal and tumour cells. Normal fibroblasts, mouse peritoneal cells and cells of low tumorigenicity infiltrated poorly and slowly whereas most tumours infiltrated rapidly. Some cells of the more invasive tumours achieved minimum rates of migration through the normal tissue of 2–3 μm/h. One tumour line which tended to form aggregates on the chorioallantoic membrane elicited a pronounced rejection response from the ectoderm of the membrane. Colcemid, which inhibits the formation of cell processes and the directional migration of cells, and dibutyryl cyclic AMP, which restores certain aspects of normal behaviour to tumour cells *in vitro*, had little effect on the invasion of the membrane by tumour cells.**

THE MECHANISMS by which tumour cells infiltrate normal host tissues are poorly understood (Easty, 1966). Various attempts to investigate the process *in vitro* have achieved some success (Easty and Easty, 1963; Barski and Wolff, 1965; Yarnell and Ambrose, 1969*a*, *b*; Latner, Longstaff and Lunn, 1971) but have not been susceptible to quantitative analysis. The methods used have frequently involved the confrontation of tumour cells with the cut edges of normal tissues. Such sites, involving both cell damage and stimulation of wound healing activity, were not ideal for the study of the invasive behaviour of tumour cells. In addition, if fragments of tumour were placed in contact with those of normal tissues, the region of greatest interest was that where necrosis due to lack of nutrients was most likely to occur.

In an attempt to avoid these problems, we have cultured as target tissues naturally occurring biological membranes, which if not more than 1 mm thick may theoretically be maintained in unlimited areas. We have also investigated the value of tubular structures, which similarly may be cultured in unlimited length if their diameter is sufficiently small. Such systems should also have the advantage that the added cell suspensions are all initially at the same easily recognizable starting line. Suspensions of normal or tumour cells were placed on the uncut surfaces of such organ cultured tissues and their ability to infiltrate assessed from histological sections in terms of the percentages of cells which penetrated the tissues and their depths of penetration.

In addition, the effects of colcemid, which depolymerizes cellular microtubules, and reduces orientated cell movements, and of dibutyryl cyclic AMP, which may restore many normal behavioural characteristics to transformed cells in monolayer culture, were examined.

### MATERIALS AND METHODS

*Culture of the chorioallantoic membrane from 12-day embryonated hen eggs.*—The shell covering the egg airspace was sterilized with ethanol and removed. The circle of shell membrane with attached chorioallantoic membrane (CAM) thus exposed was cut round the edge with scissors and removed. The egg contents were gently decanted leaving the CAM attached to the shell, from which it was carefully removed with blunt forceps and allowed to slide, ectoderm upwards, into Hepes buffered medium in a dish. The membrane was cut into circles 20–25 mm diameter, which were lifted using 2 pairs of forceps on to agar-coated expanded metal culture grids. The tissue was then gently pulled into place without undue stretching, so that there was no folds.

*Preparation of other tissues.*—Omentum from rats aged 6–8 weeks was removed by careful dissection, being kept moist with sterile saline throughout. The CAM of embryonated eggs of the Japanese quail was dissected in the same way as the hen eggs. Other membranous tissues included hamster omentum, chick embryo omentum, amnion from embryonated hen eggs and the scrotal sac from young rats. Tubular structures used were young rat trachea, cultured as rings not more than 1 mm deep, and chick embryo trachea which could be cultured as tubes of any length.

*Culture methods.*—These were a modification of the method of Trowell (1959) where tissue to be cultivated was supported on a stainless steel grid of expanded metal mesh at the interphase between the liquid culture medium and the gas phase. The grids were circular with a flat central well and a ridged edge to retain the cell suspensions added. The grids were dipped in molten 2% agar (Difco Bacto Agar) in Simm's saline solution and then placed in culture medium, which was Dulbecco's Eagle's medium and 10% foetal calf serum in 10% $CO_2$ in air, or medium 199 buffered to pH 7·4 with Hepes + 10% foetal calf serum in air.

Cell suspensions of BHK21, L, PyY, SV109, SR, P4, P4T, Harding–Passey melanoma cells and guinea-pig hepatoma growing as lines in monolayer culture were prepared by treatment with typsin or versene. Mouse peritoneal cells and ascites tumour were prepared by washing the peritoneal cavity of animals with culture media. Some of these cells were labelled with colloidal carbon (Günther Wagner) or with tritiated thymidine to facilitate identification in histological sections. The cell suspensions were washed 3 times with culture medium and the cell concentration adjusted to $10^7$/ml, and $5 \times 10^5$ cells added to each chick embryo CAM, and correspondingly smaller quantities to other tissues. In order to avoid initial loss of cells from the CAM surface the cultures were left undisturbed at room temperature for 1 hour after addition of the cell suspensions before transferring the culture to an incubator at 30 °C.

Colcemid at a final concentration of 5 μg/ml and 0·03 mmol/l dibutyryl cyclic AMP + 0·015 mmol/l testosterone propionate were added to suspensions of Harding–Passey melanoma, PyY, SV109, SR and P4T tumour cells and to the organ culture medium. The appropriate volumes of any solvents were added to control cultures.

*Evaluation of cell behaviour.*—Cultures were fixed in Carnoy's solution after periods of up to 7 days in culture. The behaviour of the added cell suspensions was evaluated by examination of stained (H. & E.) serial sections cut at rightangles to the upper surface of the tissue. The number of cells on the surface and within the tissue was counted, and the distances infiltrated by the added cells were measured from each cell to the nearest point on the initial tissue surface using a calibrated micrometer eyepiece. These measurements were made on sections from a minimum of 2 separate experiments and involved a minimum of 500 cells per experiment.

*Tumour production by injection of cell lines into animals.*—The ability of some of the cell lines used, BHK21, L, PyY, SV109 and Harding–Passey melanoma to give tumours following inoculation into suitable animals was checked at regular intervals. The BHK21 cells were always cultured from stock which had previously been cultured for no more than 70 generations and then maintained in a liquid nitrogen bank. Such cells were never used after being cultured from the frozen stock for more than 3 weeks and never gave rise to tumours following subcutaneous inoculation of $10^6$ cells into hamsters. L cells were originally derived from C3H mice (Earle *et al.*, 1943) and subcutaneous inoculation of $5 \times 10^6$ L cells into C3H mice never resulted in detectable tumour growth, but inoculation into adult thymectomized and irradiated CBA mice gave compact circumscribed tumours in 5 out of 6 animals. The tumour cell lines all gave rise to tumour growth following injection into appropriate animals.

RESULTS

*Culture of normal cells on the CAM*

The CAM of the hen egg consists of an outer layer of ectodermal cells and an inner endoderm enclosing a relatively large mesodermal layer consisting of loose connective tissue and blood vessels. The BHK21 normal hamster fibroblasts, which did not grow following subcutaneous inoculation into hamsters, attached and spread quickly on the upper surface of the ectoderm. They infiltrated the ectodermal layer very slowly (Fig. 1, 5; Table) and were never detected within the mesoderm. L cells, which also did not grow following subcutaneous inoculation into the strain of mice from which they originated, assumed an epithelioid appearance on the ectoderm and infiltrated very slowly (Table). Normal mouse peritoneal cells, labelled or unlabelled with colloidal carbon, attached poorly to the ectodermal surface and infiltrated the CAM to a greater extent than the BHK21 or L cells, but infiltration was not very extensive (Table).

FIG. 1.—Slight infiltration of CAM by normal BHK21 cells.

*Culture of tumour cells on the CAM*

The polyoma virus transformed BHK21 cells, PyY, penetrated the ectodermal layer rapidly within one day (Table) and appeared to cause some local damage to the normal cells of the target tissue. The SV40 virus transformed BHK21 cells (SV109) behaved very similarly, infiltrating rapidly and often causing local damage to the cells of the tissue. After 2 days many of these tumour cells had reached the mesoderm and were deep within the tissue (Fig. 2, 6; Table). Cells of the Harding–Passey mouse melanoma also invaded deeply into the ectoderm and mesoderm within 2–3 days (Fig. 8; Table), but, unlike the virus transformed BHK21 cells, they appeared initially to form very long cytoplasmic probes which infiltrated between the ectoderm cells (Fig. 7). They did not appear to cause any obvious damage to the cells of the normal tissue. It was possible to distinguish between 2 cell lines, P4 and P4T, originally derived from normal mouse lung tissue but now showing different degrees of tumorigenicity in mice (Barski *et al.*, 1966). The more tumorigenic cell line, P4T, contained a small but significant proportion of cells which were capable of penetrating deeply within the CAM (Fig. 4; Table), which the less tumorigenic line, P4, appeared incapable of doing (Fig. 3; Table).

Active responses of the CAM ectoderm to "foreign" tissue cells were observed unequivocally only when a line of rat fibroblasts which had been transformed with Schmidt–Ruppin virus, SR, was applied to the surface of the CAM. When these cells were applied as a single cell suspension they normally infiltrated quite actively (Table), but they were rather cohesive and frequently formed large

TABLE.—*Percentages of Cells Penetrating the CAM*

| Cells | Time (days) | Distance penetrated (μm) | | | | | | | | | | | | | | | | |
|---|---|---|---|---|---|---|---|---|---|---|---|---|---|---|---|---|---|---|
| | | 0 | 10 | 20 | 30 | 40 | 50 | 60 | 70 | 80 | 90 | 100 | 110 | 120 | 130 | 140 | 150 | 160 |
| BHK21 | 1 | 83 | 10 | 5 | 2 | 0·2 | | | | | | | | | | | | |
| | 2 | 87 | 6 | 6 | 0·5 | 0·5 | | | | | | | | | | | | |
| L | 1 | 98 | 2 | | | | | | | | | | | | | | | |
| | 2 | 87 | 9 | 2 | 1 | 1 | | | | | | | | | | | | |
| Peritoneal macrophages | 1 | 72 | 8 | 8 | 8 | 2 | 2 | | | | | | | | | | | |
| | 2 | 83 | 5 | 5 | 3 | 2 | 1 | 0·5 | 0·5 | | | | | | | | | |
| P4 | 1 | 65 | 20 | 10 | 3 | 1 | 0·5 | 0·5 | | | | | | | | | | |
| | 2 | 62 | 16 | 13 | 7 | 2 | 0·5 | 0·2 | | | | | | | | | | |
| G.P. hepatoma | 1 | 87 | 9 | 3 | 0·5 | 0·5 | | | | | | | | | | | | |
| | 2 | 70 | 20 | 6 | 3 | 0·5 | 0·5 | | | | | | | | | | | |
| | 5 | 56 | 19 | 13 | 6 | 3 | 2 | | | | | | | | | | | |
| SR | 1 | 71 | 18 | 9 | 2 | 0·2 | | | | | | | | | | | | |
| | 2 | 46 | 27 | 15 | 5 | 3 | 2 | 1 | 0·5 | | | | | | | | | |
| | 2 + colcemid | 44 | 24 | 14 | 6 | 3 | 2 | 1 | 0·5 | | | | | | | | | |
| | 5 | 37 | 19 | 14 | 8 | 5 | 4 | 3 | 3 | 1 | 2 | 2 | 1 | 1 | 2 | 1 | 0·2 | 0·2 |
| Walker | 1 | 73 | 11 | 7 | 2 | 4 | 2 | 1 | | | | | | | | | | |
| | 2 | 28 | 13 | 4 | 10 | 11 | 13 | 7 | 6 | 1 | 2 | 1 | 2 | 1 | | | | |
| P4T | 1 | 59 | 18 | 13 | 5 | 2 | 1 | 0·5 | 0·5 | | | | | | | | | |
| | 2 | 21 | 16 | 10 | 3 | 2 | 2 | 1 | 1 | 1 | 0·5 | 0·5 | 0·2 | 0·2 | | | | |
| PyY | 1 | 42 | 14 | 14 | 9 | 7 | 6 | 3 | 2 | 1 | 0·5 | 0·5 | | | | | | |
| | 2 | 28 | 10 | 9 | 9 | 13 | 10 | 7 | 6 | 2 | 2 | 2 | 2 | 1 | | | | |
| SV109 | 1 | 52 | 22 | 15 | 8 | 2 | 1 | 0·2 | | | | | | | | | | |
| | 2 | 41 | 16 | 14 | 9 | 8 | 4 | 2 | 2 | 1 | 0·5 | 0·5 | 0·5 | 0·5 | 0·5 | | | |
| H-P melanoma | 1 | 47 | 20 | 20 | 7 | 3 | 1 | 0·5 | 0·5 | 0·2 | 0·2 | 0·2 | | | | | | |
| | 2 | 33 | 13 | 11 | 8 | 7 | 7 | 4 | 4 | 4 | 3 | 2 | 1 | 1 | 2 | | | |

FIG. 2.—Extensive and progressive infiltration of CAM by SV40 virus transformed BHK21 cells.

aggregates on the CAM surface. When this occurred, the ectoderm appeared to respond by migrating towards the aggregate and extruding it into the overlying space (Fig. 9). The ectoderm was increased in thickness underneath the tumour cell aggregates and was thinner elsewhere. This response was rapid, within one day, and as no mitoses could be detected within the cells of the protruding stalk of ectodermal cells it appeared highly probable that the aggregates of SR cells had induced the migration of the ectodermal cells rather than their proliferation.

Walker carcinosarcoma cells of the rat were an example of tumour cells which appeared to adhere rather poorly to the ectoderm yet had a high infiltrative capacity (Fig. 10; Table). They also appeared to cause considerable damage to the ectodermal layer. The only carcinoma cells tested in this system were a line of guinea-pig hepatoma cells derived from a diethylnitrosamine induced tumour. These cells were rather poorly adhesive to the ectoderm and weakly infiltrative (Table), tending to invade as small aggregates rather than as single cells.

Multilayers of normal BHK21 or L cells did not appear to cause damage to the ectodermal cells, whereas even single cell layers of tumour lines such as PyY, SV109 and Walker tumour cells appeared to damage the ectoderm, presumably by the release of substances from the tumour cells. Infiltrating tumour cells were occasionally seen in blood vessels of the CAM, and this was undoubtedly one route

FIG. 3.—Moderate infiltration of CAM by mouse P4 cells of low tumorigenicity.

of invasion, but the great majority were extravascular. Significant numbers of the actively infiltrating tumours, PyY, SV109, SR, Harding–Passey melanoma and Walker tumour cells were seen after 3 days at depths of up to 200$\mu$m from the initial upper surface of the CAM, implying a minimal migration rate through the tissue of about 3 $\mu$m/hour, and almost certainly greater than this when the probably circuitous route which they had pursued is considered.

*Effects of colcemid and dibutyryl cyclic AMP*

Colcemid at 5 $\mu$g/ml was added to cultures of Harding–Passey melanoma, P4T, SR and PyY cells grown on the CAM. As reported by Vasiliev *et al.* (1970), colcemid produced retraction of many of the cell processes and stimulated the general membrane activity of these cells in monolayer culture. These effects, which were particularly marked in the Harding–Passey melanoma cells, which normally possess a number of very long processes per cell, did not significantly increase or decrease the extent of infiltration of the CAM by the cells. Dibutyryl cyclic AMP and testosterone propionate did not detectably alter the morphology of SV109, PyY and Harding–Passey melanoma cells in monolayer culture and only slightly reduced their capacity to infiltrate the CAM. The dibutyryl cyclic AMP and testosterone propionate did, however, have a very marked effect on the natural contraction of the cultured CAM. In the absence of cyclic AMP, the CAM, with or without tumour cells, contracted within 3 days to a diameter which was approximately half to one-third of the

FIG. 4.—Extensive infiltration of CAM by a small proportion of P4T cells.

original size. This contraction was uniformly and reproducibly eliminated in the presence of cyclic AMP, with the result that the upper layer of ectodermal cells was much thinner and therefore presented a weaker barrier to tumour cell infiltration.

*Other tissues*

Chorioallantoic membranes from the eggs of the Japanese quail were used as target tissues because their cells contained a region of condensed heterochromatin which facilitated identification of added mammalian cells (Le Douarin and Barq, 1969). As with the CAM of the hen egg, it was found that L cells did not significantly invade the quail CAM, whereas the highly invasive tumour cells penetrated rapidly, but the use of this tissue was abandoned because of the small areas of CAM available from each egg.

Omentum from 8-week old rats was actively invaded by PyY and SV109 cells but not by BHK21 normal fibroblasts or L cells. Its use was not pursued, however, because of its thinness and the frequent naturally occurring holes which occur in this tissue. Chick embryo omentum was of suitable thickness, lacked the holes present in rat omentum and was actively invaded by tumour cells, but it contained frequent regions of organized connective tissue which appeared to orientate the invading tumour cells, making quantitative evaluation of infiltration difficult. Scrotal sac from young rats proved to be very suitable for organ culture. Tumour cells

Fig. 5.—Normal tritium labelled BHK21 cells spread on the ectoderm of the CAM after 2 days' culture.

Fig. 6.—SV109 tumour cells, infiltrating the ectoderm and mesoderm of the CAM after 2 days' culture.

Fig. 7.—Pigmented Harding–Passey melanoma cells on the CAM after 1 day's culture showing insertion of cell processes into the normal tissue.

Fig. 8.—Harding–Passey melanoma cells penetrating deeply within the CAM mesoderm after 2 days' culture.

FIG. 9.—The response of the CAM ectoderm to aggregates of tritium-labelled SR tumour cells after 2 days' culture. The aggregates are supported on stalks composed of unlabelled ectoderm cells which have probably migrated towards and extruded the tumour aggregates.

FIG. 10.—Extensive infiltration of the CAM by Walker carcinosarcoma cells after 1 day's culture, accompanied by indications of damage to the host tissue.

FIG. 11.—SV109 tumour cells on the surface of rat scrotal sac after 5 days' culture. Note mitotic figures in the tumour layer and complete absence of infiltration.

FIG. 12.—Invasion of chick embryo trachea by SV109 tumour cells after 3 days' culture.

attached, spread and proliferated on its surface but did not infiltrate at all through the layer of dense collagen with which they were in contact over a period of 7 days' culture (Fig. 11). At the outer cut edge of the scrotal sac tumour cells had migrated from the upper surface past the cut collagen layer and then proceeded actively to infiltrate the underlying muscle tissue. Tumour cell suspensions were inoculated into the lumen of organ cultures of tracheas of 18-day chick embryos or young rats. Although these cells survived and proliferated, in no instance were any of them observed to have attached or invaded the cells lining the lumen. Subsequent observations of the organ cultures of trachea revealed that the cells lining the lumen were still indulging in active ciliary activity after 7 days of culture, and it is possible that this activity was sufficient to prevent tumour cell attachment and invasion. Very active infiltration of the trachea was observed, however, by spilt tumour cells through the cut ends and from the outside but, although tumour cells made intimate contact with the cartilage no invasion or damage to the cartilage was observed (Fig. 12), a result identical with that obtained by Dontenwill, Chevalier and Reckzeh (1973) with carcinomata induced in mice.

### DISCUSSION

The results indicated that the CAM of the embryonated hen egg was particularly suitable for the measurement of the infiltrative ability of tumour cells. Except for the outer cut edge of the CAM, which did not make contact with the added cells, this tissue provided large areas of relatively undamaged target tissue which could be maintained in culture for reasonable periods of time without loss of structure or cell viability. In addition, the added cell suspensions commenced the infiltrative process at an easily recognizable starting line which had not been subjected to surgical trauma. This considerably facilitated the measurement of cell infiltration.

The results indicate that normal cells and the cells of low tumorigenicity tested, *e.g.* BHK21, L, P4 cells, infiltrate very slowly. When slight infiltration of these cells is observed, it is usually associated with areas of minor damage to the ectoderm, probably resulting from removal from the egg (Ganote, Beaver and Moses, 1964). Unstimulated mouse peritoneal cells infiltrated slowly and were poorly adhesive to the ectoderm. This lack of infiltration of peritoneal cells would appear to support the observations of Wood, Baker and Marzocchi (1967), who found that macrophages examined by time lapse cinemicrography in rabbit ear chambers were essentially immotile. Any motility which they may demonstrate *in vivo* is probably related to response to stimuli.

Sarcoma cells infiltrated very quickly, usually as single cells, some of them achieving rates of migration through the CAM of at least 3 $\mu$m/hour, whereas the single carcinoma examined infiltrated much more slowly. Infiltration of Harding–Passey melanoma cells appeared to be associated with the ability of these cells to insert very long probes between adjacent cells of the ectoderm, but marked reduction of the lengths of the probes by treatment of the cells with colcemid did not significantly reduce their infiltrative capacity. These cells did not visibly damage the normal cells of the CAM, and preliminary electron microscopic examination of the invaded tissue confirmed this impression gained at the light microscopic level. The other tumours damaged many of the normal cells with which they made contact. This harmful effect was not observed when normal cells were placed on the CAM, even in the form of multilayers. The marked rejection of aggregates of SR cells by what is probably a migratory response of the ectodermal cells of the CAM was not observed so clearly with any other type of cell examined, and is presumably an example of a non-immune defence mechanism of the target tissue.

Dibutyryl cyclic AMP has been reported to restore many of the *in vitro* properties of normal fibroblasts to cells that have been transformed by viruses or chemicals (Johnson, Friedman and Pastan, 1971; Hsie and Puck, 1971; Macintyre *et al.*, 1972). For example, these transformed cells which have the ability to form multilayers with random cell orientation and strong agglutinability by wheat germ lipase recover many of the properties of normal fibroblasts following treatment with dibutyryl cyclic AMP, forming monolayers with parallel orientation and acquiring weak agglutinability by wheat germ lipase. Addition of dibutyryl cyclic AMP and testosterone propionate to cultures of PyY, SV109 and Harding–Passey melanoma cells on the CAM did not significantly reduce their ability to infiltrate the CAM, indicating that this property was not repressed in these tumour cells by treatment with these substances, although the normal contraction of the CAM was completely abolished. It may be significant that although Macintyre *et al.* (1972) observed that human tumour astrocytes grew more slowly and to lower terminal cell densities following treatment with dibutyryl cyclic AMP, these cells still grew in overlapping patterns and did not develop contact inhibition of movement or growth under these conditions.

Colcemid is known to change the morphology of fibroblasts, causing contraction of cytoplasmic processes and largely eliminating directional migration (Vasiliev *et al.*, 1970). Similar effects of colcemid were observed on monolayer cultures of SR, PyY and Harding–Passey melanoma cells in this work, but treatment of these cells with colcemid did not significantly inhibit their ability to infiltrate the CAM (Table).

The long cytoplasmic processes of the Harding–Passey melanoma cells, which were such a prominent feature of their infiltration between the ectodermal cells, were abolished but invasion took place just as rapidly. These long processes did not, therefore, appear to play an essential role in the invasion of these tumour cells.

### REFERENCES

Barski, G. & Wolff, E. (1965) Malignancy Evaluation of *in vitro* Transformation of Mouse Cell Lines in Chick Mesonephros Organ Cultures. *J. natn. Cancer Inst.*, **34**, 495.

Barski, G., Billardon, C., Jullien, P.-M. & Carswell, E. (1966) Evolution *in vitro* et Cancerisation des Cellules Pulmonaires de Souris. *Int. J. Cancer*, **1**, 541.

Dontenwill, W., Chevalier, H.-J. & Reckzeh, G. (1973) Growth of Carcinomas in the Region of the Cartilage. *J. natn. Cancer Inst.*, **50**, 291.

Earle, W. R., Schilling, E. L., Stark, T. H., Straus, N. P., Brown, M. F. & Shelton, E. (1943) Production of Malignancy *in vitro*. IV, The Mouse Fibroblast Cultures and Changes seen in the Living Cells. *J. natn. Cancer Inst.*, **4**, 165.

Easty, G. C. (1966) Invasion by Cancer Cells. In *Biology of Cancer*. Ed. E. J. Ambrose and F. J. C. Roe. London: Van Nostrand. p. 78.

Easty, G. C. & Easty, D. M. (1963) An Organ Culture System for the Examination of Tumour Invasion. *Nature, Lond.*, **199**, 1104.

Ganote, C. E., Beaver, D. L. & Moses, H. L. (1964) Ultrastructure of the Chick Chorioallantoic Membrane and its Reaction to Inoculation Trauma. *Lab. Invest.*, **13**, 1575.

Hsie, A. W. & Puck, T. T. (1971) Morphological Transformation of Chinese Hamster Cells by Dibutyryl Cyclic 3′ : 5′-monophosphate and Testosterone. *Proc. natn. Acad. Sci. U.S.A.*, **68**, 358.

Johnson, G. S., Friedman, R. M. & Pastan, I. (1971) Restoration of Several Morphologic Characteristics of Normal Fibroblasts in Sarcoma Cells Treated with Adenosine-3′ : 5′-cyclic Monophosphate and its Derivatives. *Proc. natn. Acad. Sci. U.S.A.*, **68**, 425.

Latner, A. L., Longstaff, E. & Lunn, J. M. (1971) Invasive Properties of Histone Transformed Cells. *Br. J. Cancer*, **24**, 568.

Le Douarin, N. & Barq, G. (1969) Sur l'utilisation des Cellules de la Caille Japonaise comme " marquers biologiques " en Embryologie Expérimentale. *C.R. Acad. Sci. Paris*, **269**, 1543.

Macintyre, E. H., Wintersgill, C. J., Perkins, J. P. & Vatter, A. E. (1972) The Responses in Culture of Human Tumour Astrocytes and Neuroblasts to $N^6$, $O^{2\prime}$-dibutyryl adenosine 3′,5′-monophosphoric acid. *J. cell Sci.*, **11**, 639.

Trowell, O. A. (1959) The Culture of Mature Organs in a Synthetic Medium. *Expl Cell Res.*, **16**, 118.

Vasiliev, J. M., Gelfand, I. M., Domnina, L. V., Ivanova, O. Y., Komm, S. G. & Olshevskaya, L. V. (1970) Effect of Colcemid on the Locomotory Behaviour of Fibroblasts. *J. Embryol. exp. Morph.*, **24**, 625.

Wood, S. Jr, Baker, R. R. & Marzocchi, B. (1967) Locomotion of Cancer Cell *in vivo* compared with Normal Cells. In *Mechanisms of Invasion in Cancer*. Ed. P. Denoix. Berlin: Springer–Verlag. p. 26.

YARNELL, M. M. & AMBROSE, E. J. (1969*a*) Studies of Tumour Invasion in Organ Culture. I. Effects of Basic Polymers and Dyes on Invasion and Dissemination. *Eur. J. Cancer*, **5**, 255.

YARNELL, M. M. & AMBROSE, E. J. (1969*b*) Studies of Tumour Invasion in Organ Culture. II. Effects of Enzyme Treatment. *Eur. J. Cancer*, **5**, 265.